# Influence of the Pulsed Voltage Connection on the Electromagnetic Distortion in Full-Size HVDC Cable PEA Measurements

**DOI:** 10.3390/s20113087

**Published:** 2020-05-29

**Authors:** Guillermo Mier Escurra, Armando Rodrigo Mor, Peter Vaessen

**Affiliations:** 1 High Voltage Laboratory, DCE&S, Department Electrical Sustainable Energy, Delft University of Technology, Mekelweg 4 2628 CD Delft, The Netherlands; A.RodrigoMor@tudelft.nl; 2 KEMA Laboratories, Klingelbeekseweg 195, 6812 DE Arnhem, The Netherlands; Peter.Vaessen@kema.com

**Keywords:** space charges, pulse electro-acoustic method (PEA), electromagnetic compatibility (EMC), high voltage cables, piezoelectric sensor

## Abstract

Nowadays, with the widespread use of High Voltage Direct Current (HVDC) cables in power systems, the measurements of space charges in full-size cables are becoming more relevant. One of the most common methods used for space charge measurements is the Pulsed Electro-Acoustic (PEA) method. This paper analyzes two factors that influence the electromagnetic interference on the piezoelectric signal. These factors are the connection of the injected pulsed voltage at the PEA test cell and the grounding of the PEA test cell. The influence was analyzed by means of experimental tests to compare different configurations and the electromagnetic distortion created in each one of them. It was observed that the physical location of the pulsed voltage at the electrode has a very important impact on the magnitude of the electromagnetic distortion. Moreover, it is shown that the physical connection of the grounding and the existence of a parasitic capacitance at the PEA test cell are also an important source of distortion.

## 1. Introduction

Space charge measurements in High Voltage Direct Current (HVDC) cables are becoming more relevant due to the increase of polymeric materials for cables in HVDC systems. As is well known, the space charge phenomenon is one of the main challenges for the development of solid HVDC dielectric systems. The presence of space charges in a dielectric distorts the electric field distribution, which may lead to accelerated aging and breakdown [1,2,3,4,5,6,7,8,9,10].

The acoustic and thermal methods are the most common non-destructive methods for measuring space charges in a dielectric [11,12,13,14,15,16,17,18,19]. Even though a lot of research had been done for the improvement of these measuring methods, most efforts have been put into flat samples and mini cables. The fact is that in power systems, the use of coaxial geometries such as in cables is more common. Moreover, successful space charge measurements had been performed since the 1990s [20,21,22]. The practice of measuring space charges in full-size cables, instead of mini cables, has the advantage of assessing the manufacturing process of cables, as well as allowing for the ability to test a combination of variables that can only be achieved in full-size cables, such as the combination of absolute temperatures and temperature gradients [23].

The aim of this paper is to analyze practical aspects of the construction of the acoustic method for space charge measurements known as the Pulsed Electroacoustic (PEA) method used in full-size HVDC cables. The paper focuses on the influence of the physical location of the voltage pulse connection in the PEA test cell electrode, as well as the influence of the test cell grounding.

The physical location of the connection has an impact on the current distributions at the PEA test cell, inducing a disturbance in the sensor-amplifier circuit. Such a spurious signal can superimpose on the useful PEA signal, which can potentially lead to incorrect post-processing and analysis. A common procedure to compensate for the effect of the voltage impulse disturbance is to use measured signals without applied direct current (DC) voltage on the sample (volt-off condition) before the sample has accumulated space charges and to subtract it from subsequent measurements by software. This procedure may prove ineffective for measurements with pre-charged samples, as subtracting the disturbance will also subtract the space charge components, as is stated in the next section of this document. Moreover, in extreme cases of pulse disturbance, the magnitude and duration of the distortion results in an effective reduction of the vertical resolution of the acoustic signal and may even result in the saturation of the amplifiers.

In this article, the effect of the pulse distortion on space charge measurements with reference to the pulsed voltage connection location and the ground connection is discussed, and test results which show the different impacts of each scenario are presented. This paper is organized as follows. Section 2 presents the theoretical background, with a brief introduction of PEA measurements and the effect of the pulse distortion. In Section 3, the test setup is described for each configuration. In Section 4, the results of the tests and the discussion are presented. Section 5 ends this paper with a conclusion.

## 2. Theoretical Background

The PEA method for the space charge measurement consists of measuring the dynamic mechanic displacement of charges in a dielectric under the effects of a pulsating electric field. The measurement is followed by a post-processing procedure to calculate the polarity, magnitude and position of the charges across the dielectric sample. The displacement force of the charges is produced by the application of a pulsating electric field across the dielectric under test. The pulsating electric field modifies the electrostatic force balance across the dielectric, as shown in [24].

(1) $$f=\rho E-\frac{\varepsilon_{0}}{2}E^{2}\nabla \epsilon_{r}-\frac{\varepsilon_{0}}{2}\nabla \left(E^{2}a\right)+\Pi \nabla E,$$

(2) $$E=E_{DC}+∆E$$

where f is the electrostatic force density, ρ is the charge density, E is the electric field strength, ε is the permittivity of the material, a is the electrostrictive coefficient and Π is the permanent dipole density. $E_{DC}$ represents the electric field strength from the DC source and ∆E the electric field strength from the voltage pulse. The transient part of f generates propagating acoustic waves, which are measured by an acoustic sensor located in one of the electrodes.

The PEA method has been successfully applied to different geometries such as DC cables, compensating for the geometric factor of the cylindrical dielectric. In full-size HVDC cables, the application of the pulsating electric field across the dielectric represents a challenge. A pulsed voltage applied directly between the inner and outer conductor of a cable needs a decoupling capacitor. For long cables, the frequency components with wavelengths smaller than the length of the cable will not perceive the cable as a lumped element and will reflect at its terminations [22,25]. For this reason, the common PEA configuration for the voltage pulse in full-size HVDC cables is an indirect injection method, using the cable as a decoupling capacitor.

**Figure 1:**
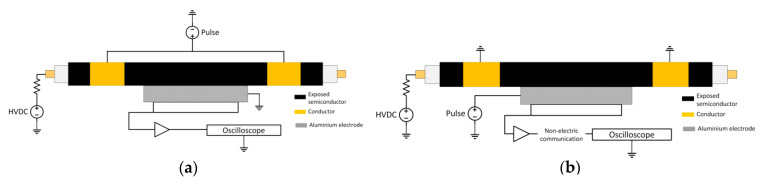
Application of the pulsed voltage. (**a**) Pulse application via the outer screen. (**b**) Pulse application via the test cell.

In Figure 1, two commonly used configurations are shown. Figure 1a represents the application of the pulse via the outer screen, which implies that the PEA cell is at ground potential, to which grounded measuring equipment can be directly connected. Figure 1b represents the situation when the pulse is applied directly at the PEA test cell, which requires that one isolate the measuring equipment from the ground, normally using an electro-optical conversion to get access to the measured signal. The impedance to the ground seen by the applied pulsed voltage can take many forms depending on the used connection, ranging from a direct ground connection to inductive values due to ground loops formed by the measuring coax cables, or even a parasitic capacitance for floated measuring equipment. This has a big impact on the disturbance at the measured signals produced by the pulsed voltage, as will be later demonstrated in this paper.

In most measurements using the PEA method, the post-processing calibration factors are obtained using a reference measured signal, whose charge values at the electrode’s interfaces are known. This is the case for a sample with an applied DC voltage when the sample is space charge free. If the sample already has space charges, meaning that the reference signal cannot be directly measured, the reference signal can be obtained by means of two measurements: The first measurement is done by applying a known DC voltage, and the second measurement is done without DC voltage, as stated in [26]. These measurements are then subtracted, obtaining the reference signal (Equation (5)):

(3) $$V_{on}\left(t\right)=Ke_{p}\left[\sigma_{1-SC}+\sigma_{2-SC}+\delta_{1}+\delta_{2}+\sigma_{1}\left(E_{DC}\right)+\sigma_{2}\left(E_{DC}\right)+v∆T\rho \left(vt\right)\right],$$

(4) $$V_{off}\left(t\right)=Ke_{p}\left[\sigma_{1-SC}+\sigma_{2-SC}+\delta_{1}+\delta_{2}+v∆T\rho \left(vt\right)\right],$$

(5) $$V_{ref}\left(t\right)=V_{on}\left(t\right)-V_{off}\left(t\right)=Ke_{p}\left[\sigma_{1}\left(E_{DC}\right)+\sigma_{2}\left(E_{DC}\right)\right],$$

where $V_{on}$ and $V_{off}$ represent the measured signal when DC is on or off, respectively. Subscripts “1” and “2” refer to the closest and farthest electrode, with respect to the acoustic sensor. $e_{p}$ is the amplitude of the pulse voltage, $\sigma_{1-SC}$ and $\sigma_{2-SC}$ are the induced charges at electrodes 1 and 2 due to the trapped space charges, $\sigma_{1}$ and $\sigma_{2}$ are the capacitive charges at electrodes 1 and 2 due to the external applied DC voltage, $\delta_{1}$ and $\delta_{2}$ are the charges at each electrode due to the applied pulse voltage, $E_{DC}$ is the external applied DC voltage, ρ is the bulk charge at the dielectric, v is the acoustic propagation speed at the dielectric, ∆T is the width of the voltage pulse and K represents the conversion factor of the acoustic sensor.

The previous procedure allows us to calibrate the measurements of a pre-charged sample, except in the case when the pulse disturbance time duration is longer than the acoustic propagation delay. In this case, the reference signal can still be obtained using the previous procedure, but it does not consider the distortion, as it gets eliminated during calculations. The problem arises when the calculated calibration factors from this reference signal are applied to the measurements with space charges and disturbance that are overlapped, as these two components cannot be separated. Equation (6) shows the calculation of the reference signal, where the distortion component named as$\;K_{dist}$ gets eliminated so that the distortion will not be compensated in subsequent calculations:

(6) $$V_{refdist}\left(t\right)=V_{ref}\left(t\right)=V_{on+dist}\left(t\right)-V_{off+dist}\left(t\right)=(V_{on}\left(t\right)+K_{dist}\left(t\right))-\left(V_{off}\left(t\right)+K_{dist}\left(t\right)\right)$$

The optimal situation is to keep the influence of the distortion $K_{dist}$ at a minimum. For this purpose, the setup explained in Section 3 is used for the experiments in Section 4. These experiments analyze the influence of the setup grounding and pulsed voltage connection towards $K_{dist}$.

## 3. Experimental Setup

### 3.1. PEA Test Cell

**Figure 2:**
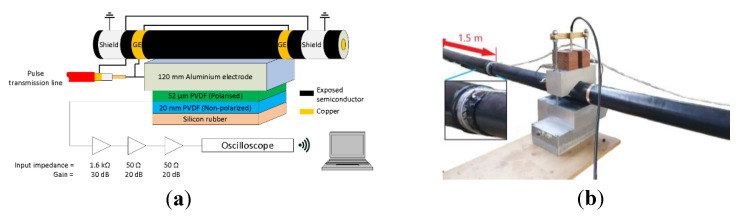
(**a**) Representation of the PEA test cell including the acoustic sensor, amplifier and oscilloscope. GE stands for guard electrode. The sizes of the components are not to scale. (**b**) PEA test cell setup.

The PEA test cell used for the experiments was built while taking into account the geometry of the cable sample. A diagram illustrating the PEA cell can be seen in Figure 2. The PEA cell uses a flat electrode configuration to facilitate an optimum acoustic contact with the cylindrical geometry of the HVDC cable. The electrode consists of a 120 mm thick aluminum block between the external semiconductor layer of the HVDC cable and the piezoelectric film. The lateral dimensions of the block are 300 mm × 300 mm. These dimensions were selected in view of the acoustic propagation speeds of 6420 m/s and 2000 m/s for the aluminum and XLPE, respectively. With a cable insulation thickness of 21.5 mm, the minimum thickness of the aluminum electrode required to avoid the overlapping of the signal due to reflections is 35 mm [27].

(7) $$d_{Al}>d_{d}\frac{\upsilon_{AL}}{2v_{d}}$$

where $d_{d}$ and $d_{Al}$ are the thicknesses of the sample dielectric and aluminum electrode, respectively. Meanwhile, $\upsilon_{d}$ and $\upsilon_{AL}$ represent the average acoustic propagation velocities of the dielectric sample material and the aluminum electrode, respectively.

The acoustic sensor consists of a 52 µm thick polyvinylidene fluoride (PVDF) piezo film, backed with 20 mm of non-polarized PVDF to avoid reflections and terminated with rubber for damping purposes. The contact area of the piezo film is 5 × 65 mm^2^, with the longest side parallel to the length of the HVDC cable. The capacitance of the piezo is 0.83 nF. The PVDF piezo film is connected to a charge amplifier with 1.6 kΩ input resistance and 30 dB gain, in series with two amplifiers of 20 dB, all of them battery powered. The piezo film and the amplifiers are contained in an aluminum box of 400 mm × 200 mm × 120 mm external dimensions and 4 mm wall thickness.

The acoustic signal coming from the amplifiers is filtered using a 10 MHz low-pass filter and then measured using a battery-powered oscilloscope with a sampling rate of 125 MS/s and 40 MHz bandwidth. The signals are then averaged 100 times.

The guard electrodes (GE) are connected to the PEA test cell through a cable attached to the mechanical pressure screws of the test cell, as can be seen in Figure 2b. The shield electrodes at each side of the PEA test cell are 1.5 m long, with an approximate capacitance towards the HVDC cable’s conductor of 390 pF each.

### 3.2. HVDC Cable under Test

For the test, a 320 kV HVDC cable was used as a test object. The cable uses copper as the inner conductor, and aluminum as the outer conductor. The dielectric material is cross-linked polyethylene (XLPE). The geometric characteristics of the HVDC cable sample are shown in Table 1.

The outer layers of the HVDC cable sample were removed in the middle of its length to expose the outer semiconductor and to mechanically fix the cable to the PEA test cell. The semiconductor is kept continuous, meaning that no section has been cut or removed to modify its electric continuity between electrodes. The outer semiconductor is in direct contact with the aluminum electrode of the PEA test cell. To ensure a good acoustic contact, silicone oil is used in the interface and compressive mechanical force is applied using screws.

### 3.3. Pulsed Voltage Circuit

**Figure 3:**
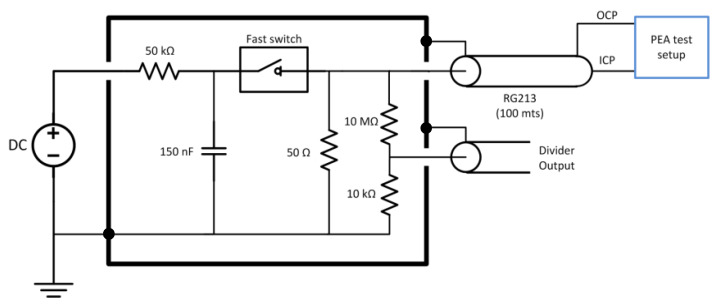
Voltage pulse generator equivalent circuit. OCP stands for the outer conductor terminal of the pulsed voltage cable at the PEA setup side, and ICP stands for the inner conductor terminal of the pulsed voltage cable at the PEA setup side.

The voltage impulse is generated using a fast switch metal-oxide-semiconductor field-effect transistor (MOSFET) Behlke HTS 61-40 using a total of 150 nF discharge capacitors and a DC source to recharge the capacitors between each pulse. The maximum charging voltage is 5.5 kV DC. The pulse travels from the switch to the PEA test cell through a coaxial cable (RG213) of 50 Ω characteristic impedance. The coax cable is 100 m long to electrically decouple the switch from the test cell due to the propagation time and the pulse duration. The pulse has a 300 ns duration. The cable is non-terminated at the PEA test setup side to maximize the voltage due to impedance mismatch and is terminated at the pulse generator side to avoid pulse reflections [28]. For future reference, in this paper, the terminal connections of the transmission line that brings the voltage pulse to the PEA test setup side are named ICP for the inner conductor and OCP for the outer conductor. The OCP conductor is grounded at the switch, as shown in Figure 3.

## 4. Test Experiments

The objective of the test experiments is to observe the impact of the pulsed voltage on the piezo-sensor distortion resulting from the current distribution across the PEA test cell during the pulse application. For this purpose, two sets of tests were performed, whose setups and results are described in Section 4.1 and Section 4.2. Each set of tests consists of several cases regarding the connection’s arrangements, described subsequently in this section.

In Section 4.1, a set of tests cell (Cases 1f, 1r, 2f, 2r, 3f and 3r) were performed to demonstrate the relevance in the selection of the pulsed voltage connection location at the PEA test cell for the generated piezo-sensor distortion. In Section 4.2, a set of tests (Cases 4f, 4r, 5f, 5r, 6f and 6r) were performed to compare and analyze the influence of the PEA test cell ground connection on the generated piezo-sensor distortion. In Section 4.3, the influence of the selected connection of the ICP terminal and the OCP terminal on the results in Section 4.1 and Section 4.2 is discussed

In the cases denoted with an “f” at the end of the case name, the pulse terminal ICP is connected to the PEA test cell and the OCP is connected to the external shield of the HVDC cable. The cases with the inverse arrangement are denoted using “r” at the end of the case name. In these cases, the OCP is connected to the PEA test cell and the ICP is connected to the external shield of the HVDC cable.

The tests were performed using the PEA test cell on the HVDC cable described in Section 3.2. This cable has been previously subjected to tests non-related to this work, in which HVDC has been applied. Due to this, the cable has pre-existing space charges which can be observed in the subsequent results. Nevertheless, the existence of space charges does not affect the results of this paper. The measurements were performed at ambient temperature without applying DC to the HVDC cable and without a temperature gradient present. For each test, the duration of the measurements was less than 1 min.

The applied voltage at the pulse circuit was 5.5 kV, from where the pulse propagated through the 50 Ω cable to reach the PEA test cell, where it was partially reflected due to the mismatching impedances (resulting in a higher applied pulsed voltage at the setup).

**Figure 4:**
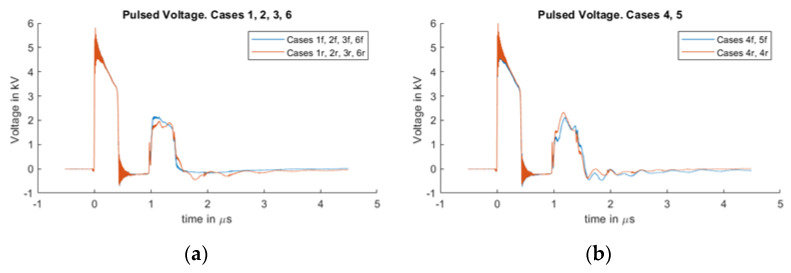
The measured voltage at the voltage pulse generator. The signal from 0 to 0.3 µs belongs to the outgoing pulse; the signal after 0.8 µs is the reflected voltage at the HVDC connection. (**a**) Measured voltage for cases 1, 2, 3 and 6. (**b**) Measured voltage for cases 4 and 5.

In Figure 4, the measured voltage at the voltage divider of the pulse generator can be observed (see Figure 3), where the initial peak belongs to the outgoing pulse of the generator and the reflected voltage waveform from the test cell arrives at 1 µs. Because the pulsed voltage at the test cell is the overlapping of the incident and reflected waves, it is possible to estimate the applied pulsed voltage by adding these two pulses. In Figure 4b, one can appreciate some oscillations at the reflected signal (after 1 µs) due to the interaction of the grounded PEA test table.

**Figure 5:**
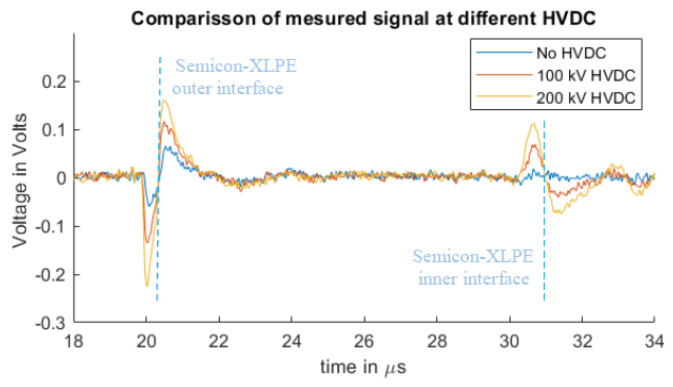
Measured signals with different applied HVDC magnitudes and a 5.5 kV pulsed voltage.

Regarding the sensitivity of the system, Figure 5 shows the measured signals with different applied DC voltages, which can be used as a reference for the sensitivity of the measuring system. The duration of the applied DC for each HVDC value was 30 s. The measured signal in the case of no voltage applied is attributed to pre-existing charges from previous HVDC with temperature tests, non-related to this work.

### 4.1. Influence of the Pulsed Voltage Connection

In this section, Cases 1f, 1r, 2f, 2r, 3f and 3r were compared to observe the influence that the physical location of the terminal connection of the pulsed voltage at the PEA test cell has on the piezo sensor distortion. Section 4.1.1 describes the setup configurations used for these tests, and in Section 4.1.2 the results and discussion are provided.

#### 4.1.1. Influence of the Pulsed Voltage Connection Test Configurations

**Figure 6:**
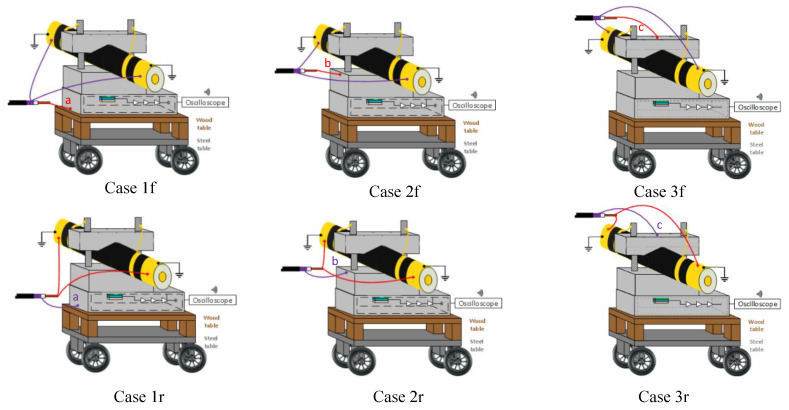
A 3D representation of the pulsed voltage connection locations for Cases 1f, 1r, 2f, 2r, 3f and 3r. Each of these cases has a different current distribution across the PEA test cell with a different impact in the piezo amplifier interference. The connection between the test cell and the guard is done through the yellow cables at the mechanical pressure screws.

For these tests, three different connection locations for the pulsed voltage at the PEA test cell were compared. Figure 6 illustrates the selected locations for the comparison used for Cases 1f, 1r, 2f, 2r, 3f and 3r. In these cases, there is a dielectric table between the metallic table and the PEA test cell to decrease the parasitic capacitance towards the ground.

- Cases 1f and 1r: Pulse injection between the base of the PEA test cell and the HVDC cable shield. The test cell is ungrounded. The HVDC cable shield is grounded.
- Cases 2f and 2r: Pulse injection between the lateral part of the PEA test, close to the upper surface of the aluminum electrode, and the HVDC cable shield. The test cell is ungrounded. The HVDC cable shield is grounded.
- Cases 3f and 3r: Pulse injection between the clamping screws of the HVDC cable to the test cell and the HVDC cable shield. The test cell is ungrounded. The HVDC cable shield is grounded.

An overview of the cases can be seen in Table 2.

#### 4.1.2. Influence of the Pulsed Voltage Connection Results and Discussion

**Figure 7:**
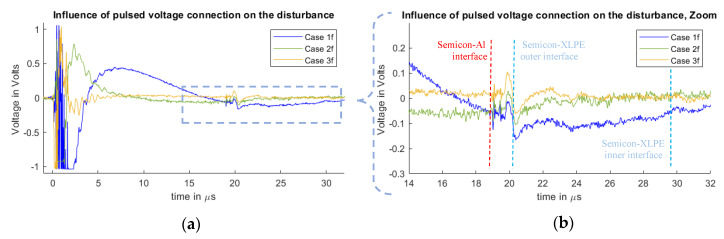
Measured disturbance from Cases 1f, 2f and 3f. (**a**) Full measured signal ranging from the instant of the pulsed voltage application up to 32 µs. (**b**) Zoom to the time instant of the acoustic signal arrival belonging to the charge measurements.

**Figure 8:**
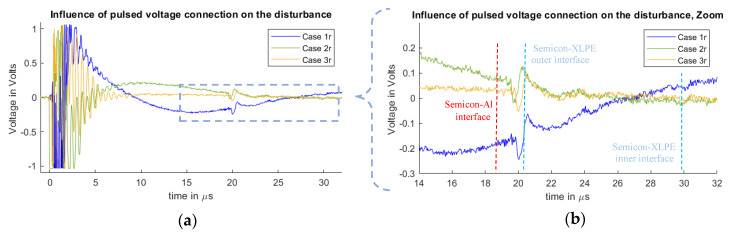
Measured disturbance from Cases 1r, 2r and 3r. (**a**) Full measured signal ranging from the instant of the pulsed voltage application up to 32 µs. (**b**) Zoom to the time instant of the acoustic signal arrival belonging to the charge measurements.

In Figure 7 and Figure 8, it is possible to observe the cases for different disturbances at the measured signals due to the connection configuration of the pulsed voltage at the PEA test cell. Figure 7 represents the measurements of Cases 1f, 2f and 3f. Figure 8 shows the results for Cases 1r, 2r and 3r, which are the inverse pulse connections.

It should be noted that the measured disturbance at the beginning of the signals (less than 1 µs) has a magnitude higher than 1 V, meaning that the amplifier saturated and that the full magnitude of the waveform cannot be observed. The main objective of this work is to compare how the disturbance reaches and overlaps with the acoustic signal belonging to the space charges region. For this purpose, the main focus is on the 19 µs time delay region.

From the measurements, one can observe the existence of space charges at the dielectric. This generates the mirror charges at the dielectric–semiconductor interfaces of the internal and external electrodes at 20 and 30 µs. The measured signal does not represent the real space charge distribution, as the signal still needs to go through post-processing to compensate for the piezo-amplifier response, geometric divergence and acoustic attenuation losses [11,12,13,14,15,16,29,30]. The spike signal that can be seen at 19 µs is generated at the vicinity of the semiconductor–aluminum interface, which is attributed to the stray capacitance between the semiconductor and the aluminum [31]. This spike was reduced for Cases 1r, 2r and 3r.

From Figure 7 and Figure 8, one can observe how the pulsed voltage injection location has a different impact on the disturbance of the signal depending on the pulse current path across the PEA test cell. When the pulse injection occurs through the base of the PEA test cell (Cases 1f and 1r), the pulse current path is closer to the amplifier, which creates a higher electromagnetic interference in comparison to Cases 2f, 2r, 3f and 3r. Between Cases 2f and 3r, as well as between 2r and 3f, the difference is not as remarkable. Nevertheless, in Figure 7b and Figure 8b, one can observe how, at the arrival time of the acoustic wave from the HVDC cable, the signal in Cases 2f and 2r is still more affected by the disturbance, adding error to the measurement.

One can note that in the measurements the disturbance reaches the relevant acoustic measurement after the delay of the aluminum electrode, Cases 1f and 1r being the worst situation.

### 4.2. Influence of the PEA Test Cell Grounding

In this section, Cases 4f, 4r, 5f, 5r, 6f and 6r were compared to observe the influence of the PEA test cell grounding on the piezo sensor distortion. Section 4.2.1 describes the setup configurations used for these tests, and in Section 4.2.2 the results and discussion are provided.

#### 4.2.1. Influence of the PEA Test Cell Grounding Test Configurations

For these tests, three different grounding configurations were measured and compared, using Cases 3f and 3r from the previous section as reference. In Cases 6f and 6r, a PEA test cell bottom surface of 230 × 330 mm is separated from a grounded surface by 20 mm of pressboard. This allows us to observe the impact on the distortion in the case of an increased parasitic capacitance towards the ground when the PEA test cell is ungrounded.

**Figure 9:**
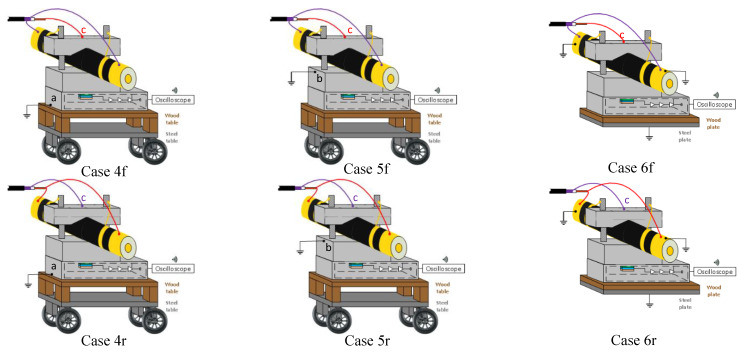
A 3D representation of the pulsed voltage connection locations for Cases 4f, 4r, 5f, 5r, 6f and 6r. Each of these cases has a different current distribution across the PEA test cell, with a different impact on the piezo amplifier interference. The connection between the test cell and the guard is done through the yellow cables at the mechanical pressure screws.

Figure 9 illustrates Cases 4f, 4r, 5f, 5r, 6f and 6r used to test the grounding effect of the test cell. In Cases 4f, 4r, 5f and 5r, the PEA test cell was grounded, each at a different location, while keeping the HVDC cable shield ungrounded. These ground connections do not represent a short circuit for the pulse circuit, as it is decoupled from the ground due to the length of the transmission line, as mentioned in Section 3.3. In Cases 4f, 4r, 5f, 5r, 6f and 6r, the location of the pulsed voltage connection at the PEA test cell is between the clamping screws of the HVDC cable (as for Cases 3f and 3r).

The cases for this test are the following:

- Cases 4f and 4r: The PEA test cell is grounded at the lower point of the aluminum block electrode. Pulse injection between the clamping screws of the HVDC cable to the test cell and the HVDC cable shield. The HVDC cable shield is ungrounded except for the pulsed voltage connection.
- Case 5f and 5r: The PEA test cell is grounded at the upper surface of the aluminum block electrode. Pulse injection between the clamping screws of the HVDC cable to the test cell and the HVDC cable shield. The HVDC cable shield is ungrounded except for the pulsed voltage connection.
- Case 6f and 6r: The test cell is ungrounded, but the extra dielectric table has been removed to increase the parasitic capacitance. Pulse injection between the clamping screws of the HVDC cable to the test cell and the HVDC cable shield. The HVDC cable shield is grounded.

An overview of the cases can be seen in Table 3.

#### 4.2.2. Influence of the PEA Test Cell Grounding Results and Discussion

**Figure 10:**
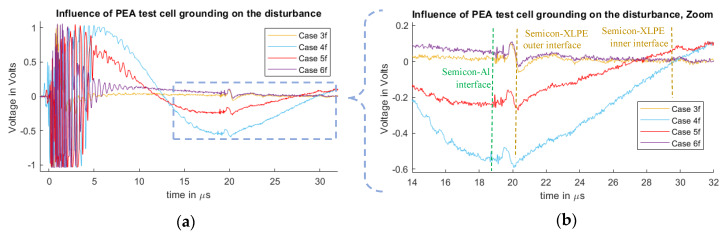
Measured disturbance from Cases 3f, 4f, 5f and 6f. (**a**) Full measured signal ranging from the instant of the pulsed voltage application up to 32 µs. (**b**) Zoom to the time instant of the acoustic signal arrival belonging to the charge measurements.

**Figure 11:**
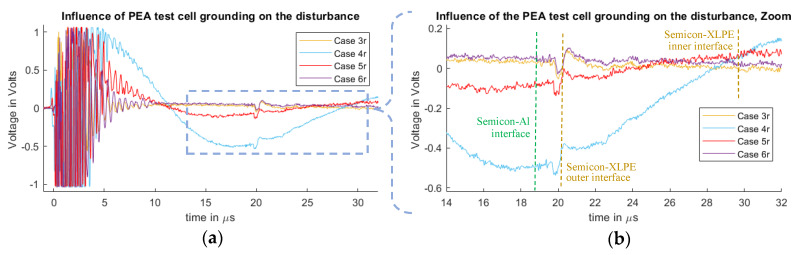
Measured disturbance from Cases 3r, 4r, 5r and 6r. (**a**) Full measured signal ranging from the instant of the pulsed voltage application up to 32 µs. (**b**) Zoom to the time instant of the acoustic signal arrival belonging to the charge measurements.

In Figure 10 and Figure 11, we can observe the measured signals from the cases 3f, 3r, 4f, 4r, 5f, 5r, 6f and 6r. Each case represents a different interaction between the PEA test cell and the ground, having an influence on the disturbance waveform at the piezo sensor. As in the previous section, the spike signal observed at 19 µs is related to the semiconductor–aluminum interface, and its effect is reduced when the pulse injection is done via the HVDC cable shield.

In Cases 3f, 3r, 6f and 6r, where the test cell is electrically isolated from the ground except from the pulsed voltage transmission cable, less distortion is present when compared to Cases 4f, 4r, 5f and 5r. This can be attributed to the extra currents at the test cell towards the ground connection, for which, in the cases 5f and 5r, the ground path is closer to the sensor.

Between Cases 3f, 3r, 6f and 6r, the difference is the higher parasitic capacitance for Cases 6f and 6r. One can observe than, in Figure 10, Case 6f is more distorted than Case 6r in Figure 11. This is attributed to the connection of the ICP terminal to the PEA test cell, where the test cell acquires a higher voltage relative to the earth during the pulse injection in comparison to the connection of the ICP terminal to the HVDC cable shield. In Figure 10b, we can observe that the difference between Case 3f and Case 6f is still visible, even after the acoustic delay. This exemplifies the relevance of the ground for the measurement setup, as the parasitic capacitance has an impact on the overall disturbance.

### 4.3. Pulsed Voltage Cable Connection Influence

In Figure 7, Figure 8, Figure 9, Figure 10 and Figure 11, the impact of the connection of the ICP and OCP to either the PEA test cell or the HVDC cable shield can be observed. Regarding the acoustic signal, the polarity is inverted, which is expected as the applied transient electric field is inverted in each configuration. Case 3 was shown to be the best result for each of the scenarios regarding the distortion. In this case, the choice of the reference (OCP) and positive (ICP) electrode does not appear to have a big impact on the quality of the signal, even in view of the fact that the shield of the HVDC cable is solidly grounded for all cases except 4f, 4r, 5f and 5r. This can be attributed to the fact that the current components of the pulse at the HVDC cable shield towards the grounding do not affect the current distribution across the PEA test cell to the same extent than in the other configurations.

## 5. Conclusions

The results of this paper, by means of experimental testing, serve as a guideline for the construction of PEA tests that minimize signal distortion and allow for simpler post-processing.

The use of a pulsed voltage in the PEA method produces an electromagnetic transient across the test cell interfering with the piezo-sensor and overlapping with the desired acoustic signal. The distortion resulting from the pulsed voltage can be substantially diminished by modifying the current distribution of the pulsed voltage across the PEA test cell in relation to the piezo amplifier position. It was observed that the physical location of the pulse voltage connection at the test cell electrode had an influence on this electromagnetic interference, measured at the piezo-amplifier circuit.

We recommend that one keep the PEA test cell isolated from the earth, even in cases where the pulsed voltage is injected through the HVDC cable shield. The existence of a grounding path creates undesired currents at the PEA test cell during the pulse injection, which can couple with the piezo-sensor and contribute to the distortion. For that reason, the use of decoupled signal acquisition devices, such as the one used in this work, is recommended.

The stray capacitance towards the PEA test cell needs to be considered, as in some cases it might raise the distortion to undesired levels.

It must be noted that the measured disturbance is dependent on the specific piezo-amplifier circuit configuration, which differs between different PEA test cell designs. Nevertheless, the measured disturbance is related to the magnitude of the interference originated by the applied voltage pulse, meaning that this paper demonstrates the influence of the connection configuration of the applied voltage pulse and the resulting magnitude of the disturbance in the piezo-amp circuit.

## Figures and Tables

**Table 1:** HVDC cable properties.

Property	Value
Inner conductor (diameter)	62.3 mm
Inner semi-conductive layer thickness	1.9 mm
Insulation thickness (XLPE)	21.5 mm
Outer semi-conductive layer thickness	1.5 mm
Exposed semiconductor length	1.5 m
Total cable length	9 m
Cable weight	34.1 kg/m

**Table 2:** Influence of the pulsed voltage connection test configurations.

Case	Pulsed Voltage Configuration		Test Cell
	ICP Connected to	OCP Connected to	
Case 1f	Test cell at point “a”	HVDC Cable shield	Ungrounded
Case 2f	Test cell at point “b”	HVDC Cable shield	Ungrounded
Case 3f	Test cell at point “c”	HVDC Cable shield	Ungrounded
Case 1r	HVDC Cable shield	Test cell at point “a”	Ungrounded
Case 2r	HVDC Cable shield	Test cell at point “b”	Ungrounded
Case 3r	HVDC Cable shield	Test cell at point “c”	Ungrounded

**Table 3:** Influence of PEA test cell grounding configurations.

**Case**	**Pulsed Voltage Configuration**		**Test Cell**
	**ICP Connected to**	**OCP Connected to**	
Case 4f	Test cell at point “c”	HVDC Cable shield	Grounded at “a”
Case 5f	Test cell at point “c”	HVDC Cable shield	Grounded at “b”
Case 6f	Test cell at point “c”	HVDC Cable shield	Ungrounded
Case 4r	HVDC Cable shield	Test cell at point “c”	Grounded at “a”
Case 5r	HVDC Cable shield	Test cell at point “c”	Grounded at “b”
Case 6r	HVDC Cable shield	Test cell at point “c”	Ungrounded
